# Cross-Cultural Adaptation and Psychometric Validation of the Polish Version of Rowland Universal Dementia Assessment Scale (RUDAS)

**DOI:** 10.3390/diagnostics15233005

**Published:** 2025-11-26

**Authors:** Monika Piotrowska-Matyszczak, Joanna Furman, Mateusz Roszak, Julia Żurawkowa, Beata Łabuz-Roszak

**Affiliations:** 1 Department of Neurology, St. Jadwiga Provincial Specialized Hospital, Institute of Medical Sciences, University of Opole, 45-221 Opole, Poland; monika.piotrowska.matyszczak@gmail.com; 2 Department of Environmental Health Risk Factors, Faculty of Public Health in Bytom, Medical University of Silesia in Katowice, 41-902 Bytom, Poland; jfurman@sum.edu.pl; 3 Student Scientific Association at the Department of Neurology, Institute of Medical Sciences, University of Opole, 45-221 Opole, Poland; mateuszroszakmail@gmail.com; 4 Neurology Department with Stroke Unit, T. Marciniak Lower Silesian Specialist Hospital-Emergency Medicine Center, 54-049 Wroclaw, Poland; juliazuraw13@gmail.com

**Keywords:** Rowland Universal Dementia Assessment Scale, Mini-Mental State Examination, dementia, cross-cultural adaptation, validation

## Abstract

**Background**: There is a growing demand for sensitive and accurate screening tools for the early detection of cognitive impairment. The Rowland Universal Dementia Assessment Scale (RUDAS) has shown promise in multicultural populations. It may offer advantages over the widely used Mini-Mental State Examination (MMSE), which has limited sensitivity, particularly in assessing executive functions. The aim of this study was to adapt and validate the Polish version of the RUDAS and to compare its diagnostic performance with the MMSE in detecting cognitive impairment among patients with Alzheimer’s disease (AD) and vascular dementia (VaD). **Methods**: A total of 126 subjects were evaluated, including 37 with AD, 30 with VaD, and 59 healthy controls. All participants were assessed with both the MMSE and RUDAS, and their test results were subsequently compared. **Results**: A strong correlation was found between total scores on the RUDAS and MMSE (RS = 0.81, *p* < 0.001). The area under the ROC curve was slightly higher for RUDAS (AUC = 0.94) than for MMSE (AUC = 0.89), suggesting better diagnostic accuracy. At a cut-off score of 25, RUDAS showed a sensitivity of 0.84 and a specificity of 0.87; MMSE showed a sensitivity of 0.74 and a specificity of 0.91. **Conclusions**: The Polish version of RUDAS demonstrates strong diagnostic utility and may offer a slightly more sensitive alternative to MMSE for dementia screening, especially in its early stages. Further normalization studies on larger and more diverse clinical populations are recommended.

## 1. Introduction

It is estimated that by 2050, the number of individuals with dementia worldwide will exceed 131 million [1].

According to the 2018 report of the United Nations Department of Economic and Social Affairs, for the first time in human history, the number of individuals aged 65 years and older exceeded the number of children under the age of five [2]. Population aging entails profound social, economic, and healthcare consequences [3]. Among the major challenges faced by older adults is cognitive decline, which in many cases may progress to neurocognitive disorders. It is estimated that approximately 600,000 people in Poland are living with dementia. In contrast, the number of individuals formally registered within the healthcare system with a diagnosis of Alzheimer’s disease and related disorders amounts to 382,600 [4].

Dementia is a clinical diagnosis established through a comprehensive evaluation that includes physical, neurological, and psychiatric examinations, laboratory testing of blood and cerebrospinal fluid, neuroimaging, neuropsychological assessments, and collateral history obtained from relatives [5]. The demand for valid and sensitive screening tools enabling the early recognition of cognitive impairment is steadily increasing, as early detection is crucial for planning further diagnostic and therapeutic strategies.

Screening of cognitive functioning is an essential step in clinical practice, as it not only objectifies and complements information obtained from patient and family interviews but also provides clinically relevant data for subsequent diagnostic procedures [6].

The most frequently used screening instrument in Europe, including Poland, is the Mini-Mental State Examination (MMSE), originally developed for detecting cognitive impairment in Alzheimer’s disease. However, it lacks sufficient sensitivity for identifying executive dysfunction [7]. Additionally, the MMSE has been adapted and validated for the Polish population.

An attempt was also made to adapt the Test Your Memory (TYM), which has established normative data; however, the validation study included only patients with Alzheimer’s disease (AD) and mild cognitive impairment (MCI) [8]. The TYM test also presents certain limitations, as it is performed independently by the participant under minimal supervision, which hinders full observation of task execution—an element that is often crucial for accurate clinical interpretation and final diagnostic conclusions.

The Rowland Universal Dementia Assessment Scale (RUDAS) [9] was developed in Australia and has shown strong psychometric properties in detecting dementia, with the advantage of being designed for use across culturally and linguistically diverse populations [10]. Furthermore, RUDAS has been validated in several high-, middle-, and low-income countries across different languages, cultures, and educational backgrounds [11,12,13]. Importantly, it also assesses frontal lobe functions (semantic fluency, judgment), which are particularly relevant in non-Alzheimer’s neurodegenerative disorders.

In Poland, there is still a shortage of standardized screening tools for assessing cognitive function in patients with suspected early neurodegenerative processes. This issue is particularly important, as access to neuropsychologists specializing in the diagnosis and differential assessment of neurodegenerative disorders remains limited. Although there are currently more and more biomarkers (fluid and neuroimaging) of neurodegeneration, neuropsychological testing is still the fastest and cheapest method, especially in the context of screening. Therefore, the aim of the present study was to adapt and validate the Polish version of the Rowland Universal Dementia Assessment Scale (RUDAS) and to compare its diagnostic performance with that of the Mini-Mental State Examination (MMSE) in detecting cognitive impairment among patients with Alzheimer’s disease (AD) and vascular dementia (VaD).

## 2. Materials and Methods

The study was conducted between July 2023 and January 2025. It was approved by the Bioethics Committee of the University of Opole, Poland, and carried out in accordance with the Declaration of Helsinki (Statement No. L.dz. EZD/183673/2023). All participants received both oral and written information about the study and subsequently provided written informed consent.

A total of 126 individuals were enrolled, including 67 patients diagnosed with Alzheimer’s disease (AD) or vascular dementia (VaD) and 59 cognitively healthy controls who did not report any memory or cognitive difficulties. Patients in the control group were admitted to the outpatient clinic or hospital ward due to other medical conditions (e.g., spinal degeneration, dizziness, etc.). Inclusion criteria were age ≥60 years, functional hearing and vision, or, when necessary, the use of appropriate and well-functioning technical aids (e.g., hearing aids, optical lenses). Study participants were recruited from outpatients of the Neurology Clinic and inpatients of the Neurology Department at the St. Jadwiga Provincial Specialist Hospital in Opole. All individuals were residents of the Opole, Silesia, or Lower Silesia Provinces.

For methodological purposes, patients with AD and VaD were treated as a single study group. Screening tests were administered in a fixed order: first the MMSE, followed by the RUDAS after a 30 min interval. The break was introduced to minimize the risk of interference with memory traces. Both tests were administered by the research group (authors 1, 3, and 4), while interpretation and evaluation of the results were performed by author 1.

Clinical diagnoses of AD and VaD were established based on physical, neurological, and laboratory examinations, brain imaging, neuropsychological testing, and collateral history from relatives, in accordance with established diagnostic criteria for dementia (American Psychiatric Association) [14] and the International Classification of Diseases (ICD-10) [15].

For the assessment of cognitive functioning, the Polish version of the MMSE [16] and a Polish translation of the RUDAS were used. RUDAS was translated into Polish by two independent translators: non-medical and medical. Both translators were bilingual native Polish speakers. The translation of RUDAS was performed in accordance with the principles of cross-cultural adaptation. Permission for use of the instrument was obtained from the original author. The translation and cross-cultural adaptation process followed the “Guidelines for the process of cross-cultural adaptation of self-reported measures” recommended by Beaton et al. [17]. The Polish version of RUDAS is attached as a Supplementary Material.

Prior to the study, a pilot version of the Polish RUDAS was administered to 10 individuals (3 with AD, 3 with VaD, 2 with mild cognitive impairment [MCI], and 2 healthy controls). No difficulties in administration or comprehension were reported, and no modifications to the task structure or format were required.

The MMSE was used as a reference point for diagnostic accuracy. The test consists of subtests assessing orientation in time and place, memory (immediate and delayed recall), working memory (serial sevens), language (naming, repetition, following a verbal command, writing), and visuospatial skills (copying intersecting pentagons). The maximum score is 30, with administration taking approximately 10 min [7].

The RUDAS consists of subtests assessing memory (immediate and delayed recall), visuospatial orientation (body schema), visuospatial skills (cube copy), praxis (alternating hand movements after demonstration), judgment and reasoning, and semantic fluency. The maximum score is 30, with administration also taking about 10 min [9].

Statistical analysis was performed using Statistica 13.3 (StatSoft Polska Sp. z o.o, Kraków, Poland). Data were presented in numerical and percentage notation. Statistical significance was determined at *p* < 0.05. Continuous variables were compared using Student’s *t*-test. Correlations between MMSE and RUDAS were analyzed using Spearman’s correlation. To compare the diagnostic accuracy of MMSE and RUDAS for dementia, ROC curve analysis was performed with clinical dementia diagnosis as the reference standard. An area under the curve (AUC) of 0.9–1.0 was considered excellent, 0.8–0.9—good, 0.7–0.8—fair, 0.6–0.7—poor, and 0.5–0.6—unsatisfactory [18].

## 3. Results

### 3.1. Sample Characteristics

In total, 126 participants were examined: 30 with VaD, 37 with AD, and 59 cognitively healthy controls. Participants ranged in age from 55 to 94 years, and there were 35 women (52.2%) and 32 men (47.8%). The mean age was 76.3 ± 7.5 years in the group with dementia and 73.2 ± 7.9 years in the group without dementia, while the average years of education were 12.5 ± 2.9 and 13.8 ± 2.9, respectively. Although the mean age values in both groups appear relatively similar, this difference was statistically significant (*p* < 0.001). A similar pattern was observed for education: despite the seemingly modest difference in the average years of schooling, this difference was also statistically significant (*p* < 0.001).

Demographic and clinical data, as well as test results (MMSE and RUDAS), are presented in Table 1.

### 3.2. Correlation

The overall results of the MMSE and RUDAS in our study demonstrated a strong and statistically significant correlation. Spearman’s correlation coefficient was (Rs = 0.81, *p* < 0.001) when analyzing the scores of all participants. There was also a significant correlation between the MMSE score and age (Rs = −0.25, *p* = 0.005), between the RUDAS score and age (Rs = −0.34, *p* < 0.001), MMSE score and years of education (Rs = 0.33, *p* < 0.001), RUDAS score and years of schooling (Rs = 0.29, *p* < 0.001).

### 3.3. Diagnostic Accuracy and Optimal Cutoff Values

Table 2 and Table 3 present the diagnostic accuracy of MMSE and RUDAS for various cutoff scores in screening for dementia (AD + VaD).

MMSE achieved optimal discrimination of dementia patients at a cut-off score of ≤25, with a sensitivity of 0.82 (95% CI 0.73–0.91) and a specificity of 0.95 (95% CI 0.89–1.00). RUDAS achieved optimal discrimination at a cut-off score of ≤24, with a sensitivity of 0.82 (95% CI 0.73–0.91) and a specificity of 0.93 (95% CI 0.87–1.00). While both tests demonstrated identical sensitivity, MMSE showed slightly higher specificity. Combined with a higher positive likelihood ratio (LR+) of 16.14 versus 12.11 and a positive predictive value (PPV) of 0.95 versus 0.93, this suggests a modest advantage of MMSE over RUDAS. Nevertheless, these findings indicate comparable diagnostic accuracy, supporting the interchangeability of both screening instruments.

**Figure 1:**
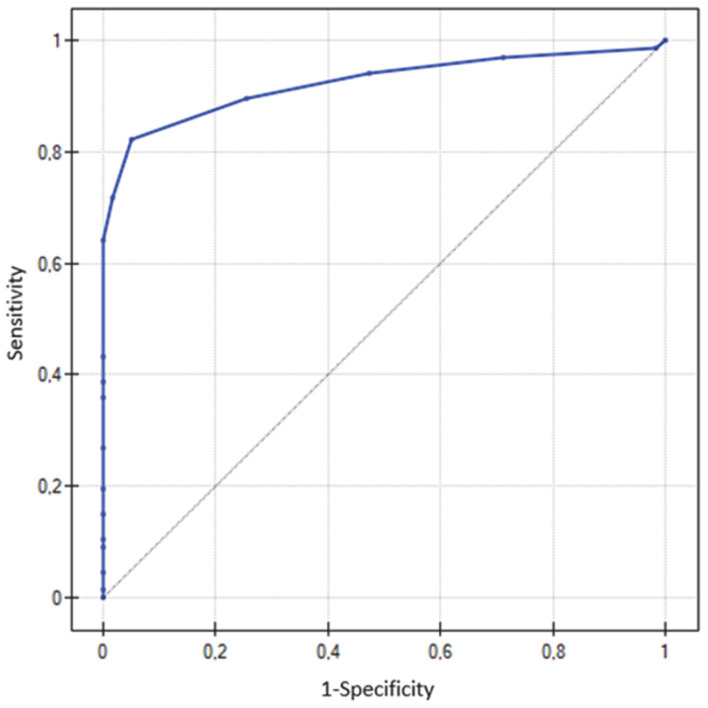
ROC curve for the MM SE test, AD + VaD; AUC = 0.92 (95% CI: 0.87–0.97). The diagonal line represents the random classifier.

**Figure 2:**
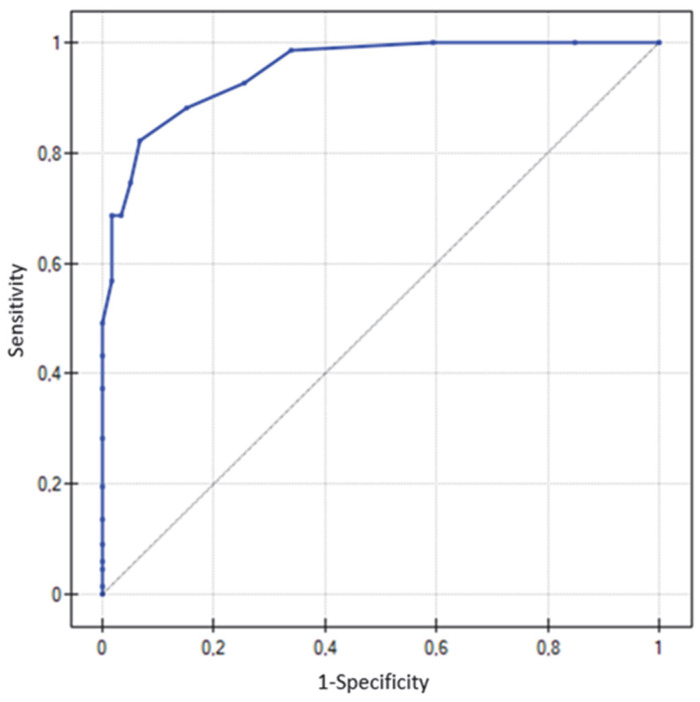
ROC curve for the RUDAS test, AD + VaD; AUC = 0.95 (95% CI: 0.92–0.98). The diagonal line represents the random classifier.

ROC curve analyses were conducted separately for MMSE and RUDAS (Figure 1 and Figure 2). The AUC for RUDAS was 0.95 (95% CI: 0.92–0.98), and for MMSE 0.92 (95% CI: 0.87–0.97), indicating that both tests discriminated with good accuracy between dementia patients and cognitively healthy individuals. Overall, RUDAS showed a slightly higher AUC compared with MMSE, suggesting marginally greater diagnostic accuracy in detecting cognitive impairment.

## 4. Discussion

In the present study, the diagnostic accuracy of the RUDAS test was evaluated in a group of patients diagnosed with Alzheimer’s disease (AD) and vascular dementia (VaD), with a control group consisting of individuals without cognitive deficits. To our knowledge, this is the first evaluation of the RUDAS test in Poland, conducted by a team of hospital professionals routinely involved in the diagnosis of dementing disorders, well-acquainted with relevant medical procedures, and possessing long-standing clinical experience.

Polish society is characterized by negative demographic growth and an accelerated process of population aging. Rapid screening for neurodegenerative disorders is becoming an increasingly pressing challenge for the overburdened healthcare system, which continues to face a shortage of specialized personnel. Therefore, the development and implementation of new, efficient diagnostic tools is particularly desirable. Our findings indicate that RUDAS demonstrates both good sensitivity and specificity in detecting cognitive impairment.

Given that the MMSE is the most widely used screening tool for dementia worldwide, and that normative studies of this test have been conducted in Poland, a comparison with RUDAS was considered justified. This approach is consistent with numerous validation studies [19,20,21,22,23,24,25,26].

In the literature, there are multiple reports on the validation of the RUDAS. Torkpoor et al. [22] examined 123 patients, comparing the diagnostic properties of RUDAS and MMSE using ROC curve analysis. The obtained AUC values were similar and judged to be good (RUDAS: 0.81; MMSE: 0.79). At an optimal cutoff score of <25, RUDAS showed a sensitivity of 0.92, specificity of 0.60, and accuracy of 76%. The authors concluded that the high sensitivity of RUDAS makes it a valuable tool for detecting dementia.

In the study by Goudsmit et al. [25], which included 152 geriatric patients of non-European origin, the authors hypothesized that RUDAS would be more accurate in detecting cognitive impairment compared to MMSE and less influenced by educational level or illiteracy. The results partially confirmed these assumptions: the AUC for RUDAS was 0.81 in the entire group and 0.89 after excluding patients with MCI, whereas the AUC for MMSE was 0.77 and 0.85, respectively. The confidence intervals for the AUC of both tests overlapped, preventing a definitive conclusion as to which test better discriminates between patient groups. Importantly, unlike MMSE, RUDAS scores were not correlated with education level or illiteracy.

Similarly, Beniam et al. [13] assessed the psychometric properties of RUDAS, demonstrating good internal consistency (Cronbach’s α = 0.73). At a cutoff score of ≤22, RUDAS showed very good ability to detect advanced neurocognitive disorders (AUC = 0.87; sensitivity = 92%; specificity = 75%). In this study, RUDAS scores were positively correlated with years of formal education.

In the study by Limpawattana et al. [26], the performance of RUDAS and MMSE was analyzed among geriatric patients. Both tests demonstrated comparable accuracy (AUC = 0.81) and strong intercorrelation (Pearson’s r). The suggested cutoff score for both instruments was ≤24. The authors emphasized that although MMSE remains the most widely used screening tool in Thailand, its limitations include variability in sensitivity and specificity, dependence on educational level, cultural and linguistic background, age, as well as time requirements and incomplete assessment of certain cognitive domains. RUDAS, less affected by these factors, may thus represent a valuable alternative screening instrument in geriatric populations.

In the present study, all participants (*n* = 126) completed both tests, including 67 patients with AD or VaD and 59 individuals from the control group. The analysis demonstrated a strong correlation between MMSE and RUDAS scores (Spearman’s ρ = 0.81; *p* < 0.001), consistent with findings from other studies [18,19]. ROC curve analysis confirmed high diagnostic accuracy for both tests (RUDAS: AUC = 0.95; MMSE: AUC = 0.92), with values slightly higher than those reported in most validation studies conducted in high-income countries in mono- or multicultural clinical samples [18,19,20,21]. This high diagnostic performance may be explained by the absence of patients with mild cognitive impairment (MCI), who were deliberately excluded. MCI patients occupy a borderline state between dementia and non-dementia, and their inclusion could have lowered ROC values. Additionally, the relatively high proportion of individuals with higher education in the sample may have elevated the normative distribution compared with the general population, potentially affecting results. An increase in ROC values after excluding patients with MCI has been observed, for example, in validation studies of the RUDAS test in Sweden [21].

For a useful cognitive screening test, high sensitivity is prioritized alongside strong overall accuracy. The high sensitivity of the RUDAS observed in this study (0.82 at a cut-off score <24), similar to that of the MMSE (0.82 at a cut-off score <25), indicates that RUDAS is a valuable tool for dementia screening. Its strong sensitivity makes it particularly suitable for the early identification of cognitive impairment in clinical practice, enabling healthcare professionals to efficiently select patients who may require further diagnostic evaluation and intervention. However, due to its slightly lower specificity (0.93 at a cut-off score <24 versus 0.95 at a cut-off score <25 for MMSE), clinicians should be aware of a potentially higher rate of false positives.

In parallel with the development of cognitive screening tools, intensive research is being conducted on metabolic biomarkers of dementia, which are intended to complement rather than replace traditional neuropsychological diagnostics. Studies by Socha et al. suggest that alterations in the amino acid profile may reflect early neurodegenerative changes and accompany cognitive decline even before the full clinical manifestation of dementia. In their paper, the authors demonstrated that serum levels of serine, arginine, and isoleucine differentiate individuals with dementia from cognitively healthy older adults, indicating the potential of these compounds as biochemical indicators [27]. In a subsequent study, the same research group described amino acid profile alterations along the continuum from mild cognitive impairment (MCI) to dementia, supporting the concept of combining biological markers with cognitive screening [28]. Together, these findings emphasize that metabolic biomarkers and cognitive assessment form a complementary ecosystem for the early detection and monitoring of dementia.

## 5. Limitations of the Study

This study has some limitations. First, the sample size was relatively small and included only patients with the most common types of dementia (AD and VaD). In the future, it will be necessary to extend validation studies to other neurodegenerative disorders, including Parkinson’s disease (PD), atypical parkinsonian syndromes, and frontotemporal dementia (FTD) with its subtypes, in order to obtain more comprehensive psychometric characteristics and to enable more detailed international comparisons.

Second, the study was conducted within a limited geographical area (Opole, Silesia, and Lower Silesia Provinces). Including patients from other regions of Poland would increase sample diversity and could allow for a more precise determination of the RUDAS cutoff score.

Third, future studies should consider including immigrant populations, particularly from Ukraine, who constitute a significant segment of society but are currently infrequently referred for cognitive screening due to the lack of culturally and linguistically adapted assessment tools.

Fourth, a potential limitation of this study is the relatively high proportion of individuals with higher education in the control group, which, combined with the limited representation of participants with only primary education (and thus fewer years of formal schooling), may have influenced test performance and affected the determination of the optimal RUDAS cutoff score.

Fifth, a potential limitation is the order of test administration (MMSE before RUDAS), which may introduce a subtle practice effect.

## 6. Conclusions

Preliminary adaptation and validation of RUDAS in the Polish population suggest that it may be considered a valuable screening tool for dementia detection. A noteworthy observation was that participants experienced lower emotional distress during RUDAS administration compared with MMSE, which may enhance its clinical applicability. This likely reflects patients’ subjective perception of the absence in RUDAS of questions related to allopsychic orientation, which are integral to MMSE and, among higher-functioning individuals, may provoke frustration and introduce distraction during test performance.

An additional advantage of RUDAS is its applicability across different professional groups without formal licensing restrictions. Although correct administration and interpretation require appropriate clinical knowledge and skills, these can be acquired through brief training provided by a neuropsychologist. These features may support the broader use of RUDAS in cognitive screening, facilitating earlier detection of neurodegenerative diseases and faster referral for diagnostic evaluation.

Taken together, these findings indicate that RUDAS can serve as a valuable supplementary tool in clinical practice for the screening and early detection of cognitive impairment.

## Figures and Tables

**Table 1:** Demographic and clinical data of examined subjects (*n* = 126).

	All the Subjects	Subjects with Diagnosed Dementia (VaD or AD)	Subjects without Diagnosed Dementia (Control Group)	*p*-Value *
Number of subjects, *n*	126	67	59	
Age, years [X ± SD]	72.8 ± 8.1	76.3 ± 7.5	73.2 ± 7.9	<0.001 ^a^
**Gender**				
Women, *n* (%)	75 (57.9%)	35 (52.2%)	40 (67.8%)	*p* = 0.076 ^b^
Men, *n* (%)	51 (40.5%)	32 (47.8%)	19 (32.2%)	
**Years of education**				
[X ± SD; minimum-maximum]	13.1 ± 3.0; 8–18	12.5 ± 2.9; 8–18	13.8 ± 2.9; 8–18	<0.001 ^a^
**MMSE**				
Total score, points [X ± SD; minimum-maximum]	24.6 ± 4.1; 13–30	22.0 ± 3.9; 13–30	27.5 ± 1.3; 24–30	<0.001 ^c^
**RUDAS**				
Total score, points [X ± SD; minimum-maximum]	23.3 ± 5.4; 10–30	19.6 ± 4.5; 10–28	27.6 ± 2.1 20–30	<0.001 ^c^

*n*—number of participants; X—mean; SD—standard deviation; AD—Alzheimer disease; VaD—vascular dementia; MMSE—Mini Mental State Examination; RUDAS—Rowland Universal Dementia Assessment Scale; ^a^ Student’s *t*-test; ^b^ Chi-square test; ^c^ Mann–Whitney U.; * comparison of groups with and without dementia.

**Table 2:** Diagnostic accuracy of MMSE in detecting (AD + VaD) at different cut-off points.

Cut-Off	Sensitivity	Specificity	PPV	NPV	LR+	LR-	A	Youden Index
22	0.43	1.00	1.00	0.61	NA	0.57	0.70	0.43
23	0.64	1.00	1.00	0.71	NA	0.36	0.81	0.64
24	0.72	0.98	0.98	0.75	42.27	0.29	0.84	0.70
25 *	0.82	0.95	0.95	0.82	16.14	0.19	0.88	0.77
26	0.90	0.75	0.80	0.86	3.52	0.14	0.83	0.64

Abbreviations: PPV—positive predictive value; NPV—negative predictive value; LR+—positive likelihood ratio; LR-—negative likelihood ratio; A—accuracy. Note: * indicates the optimal cut-off point

**Table 3:** Diagnostic accuracy of RUDAS in detecting (AD + VaD) at different cut-off points.

Cut-Off	Sensitivity	Specificity	PPV	NPV	LR+	LR-	A	Youden Index
22	0.69	0.97	0.96	0.73	20.25	0.32	0.82	0.65
23	0.75	0.95	0.94	0.77	14.68	0.27	0.84	0.70
24 *	0.82	0.93	0.93	0.82	12.11	0.19	0.87	0.75
25	0.88	0.85	0.87	0.86	5.77	0.14	0.87	0.73
26	0.93	0.75	0.81	0.90	3.64	0.10	0.84	0.67

Abbreviations: PPV—positive predictive value; NPV—negative predictive value; LR+—positive likelihood ratio; LR-—negative likelihood ratio; A—accuracy. Note: * indicates the optimal cut-off point

## Data Availability

The data presented in this study are available on request from the corresponding author. The data are not publicly available due to privacy reasons.

## Supplementary Materials

The following supporting information can be downloaded at: https://www.mdpi.com/article/10.3390/diagnostics15233005/s1, File S1: Polish version of RUDAS.

## Abbreviations

The following abbreviations are used in this manuscript:

A	Accuracy
AD	Alzheimer’s disease
AUC	Under the curve
LR-	Negative likelihood ratio
LR+	Positive likelihood ratio
MCI	Mild cognitive impairment
MMSE	Mini-Mental State Examination
NPV	Negative predictive value
PPV	Positive predictive value
RUDAS	Rowland Universal Dementia Assessment Scale
VaD	Vascular dementia
