# Supplementary material for: Cross-Cultural Adaptation and Psychometric Validation of the Polish Version of Rowland Universal Dementia Assessment Scale (RUDAS)
Source: Diagnostics (Basel). 2025 Nov 26;15(23):3005. doi: 10.3390/diagnostics15233005 (PMC12691062; doi:10.3390/diagnostics15233005)

R U D A S

The Rowland Universal Dementia Assessment Scale: A Multicultural Cognitive Assessment Scale.
(Storey, Rowland, Basic, Conforti & Dickson, 2004). International Psychogeriatrics, 16 (1), 13-31

Data: ____/____/____ Imię i nazwisko pacjenta: ______________________________

Zakres badania		Wynik
**Pamięć**<br><br>1. (Instrukcja) Wyobraź sobie, że idziemy na zakupy. Oto lista artykułów spożywczych. Chciałbym, abyś zapamiętał następujące rzeczy, które musimy kupić. Kiedy dotrzemy do sklepu za około 5 minut zapytam Cię, co musimy kupić. Zapamiętaj, co jest na liście. **Herbata, olej, jajka, mydło.** Proszę powtórz mi tę listę (poproś badanego o powtórzenie listy 3 razy). (Jeśli badany nie powtórzył wszystkich czterech słów, powtarzaj listę, aż badany zapamięta jej elementy i będzie mógł je powtórzyć, ale nie więcej niż pięć razy).		
**Orientacja wzrokowo-przestrzenna**<br><br>2. Poproszę Cię o wskazanie/pokazanie mi różnych części ciała. *(Prawidłowa odpowiedź =1)*. Kiedy badany poprawnie odpowie na 5 części tego pytania, nie kontynuuj, ponieważ maksymalny wynik to 5.		
(1) pokaż mi swoją prawą stopę	1	
(2) pokaż mi swoją lewą rękę	1	
(3) prawą ręką dotknij swojego lewego ramienia	1	
(4) lewą ręką dotknij swojego prawego ucha	1	
(5) wskaż moje lewe kolano	1	
(6) wskaż mój prawy łokieć	1	
(7) swoją prawą ręką wskaż moje lewe oko	1	
(8) swoją lewą ręką wskaż moją lewą stopę	1	**...../5**
**Praksja**<br><br>3. Pokażę Ci pewne ćwiczenie z użyciem rąk. Obserwuj mnie i naśladuj moje ruchy. Rób to samo co ja ... (Jedna dłoń zaciśnięta w pięść, druga dłoń płasko na stole – wykonuj ruch naprzemiennie).<br>Zrób to ze mną: Teraz chciałbym, abyś wykonywał tę czynność w takim tempie, aż do momentu, kiedy powiem Ci, że masz przestać - około 10 sekund. (Zademonstruj		

w umiarkowanie wolnym tempie).<br>Skala wyników:<br><br>*W normie = 2 (bardzo niewiele błędów, jeśli w ogóle; badany potrafi się sam skorygować, stopniowo osiąga coraz lepszy wynik; dobre utrzymanie; jedynie bardzo niewielki brak synchronizacji między rękami)*<br><br>*Częściowo odbiegający od normy = 1 (zauważalne błędy z pewnymi próbami autokorekty; pewne próby utrzymania; słaba synchronizacja)*<br><br>*Poniżej normy = 0 (badany nie potrafi wykonać zadania; brak utrzymania; brak jakiejkolwiek próby)*		…./2
**Rysunek wizualno-konstrukcyjny**<br><br>4. Proszę jak najdokładniej przerysować ten obrazek (Pokaż kostkę na odwrocie strony). (Tak = 1)<br>Oceń następujące elementy:		
(1) Czy badany narysował obrazek na podstawie kwadratu?	1	
(2) Czy wszystkie wewnętrzne linie pojawiają się na rysunku badanego?<br>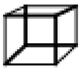	1	
(3) Czy wszystkie linie zewnętrzne są widoczne na rysunku badanego?<br>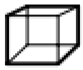	1	…./3
**Podejmowanie decyzji**<br><br>5. Stoisz na poboczu ruchliwej ulicy. Nie ma tam przejścia dla pieszych ani sygnalizacji świetlnej.<br>Powiedz, co byś zrobił, aby **bezpiecznie** przejść na drugą stronę ulicy. (Jeśli badany udzieli niepełnej odpowiedzi, która nie odnosi się do obu części odpowiedzi, użyj podpowiedzi: "Czy jest coś jeszcze, co byś zrobił?").<br>Zapisz dokładnie to, co mówi badany i zakreśl wszystkie części odpowiedzi, do których był nakłaniany.<br>……….<br>……….<br><br>Kryteria oceny:		
Czy badany wspomniał, że rozejrzy się, czy nic nie nadjeżdża? (TAK = 2; TAK, PO UDZIELENIU PODPOWIEDZI = 1; NIE = 0)	2	
Czy badany przedstawił jakieś dodatkowe propozycje dotyczące bezpieczeństwa? (TAK = 2; TAK, PO UDZIELENIU PODPOWIEDZI = 1; NIE = 0)	2	

		**…./4**
**Przypominanie**		
1. (Przypominanie) Właśnie dotarliśmy do sklepu. Czy pamiętasz listę artykułów spożywczych, które musimy kupić?<br>(Wskazówka: Jeśli badany nie może przypomnieć sobie żadnej pozycji z listy, powiedz "Pierwszą rzeczą była 'herbata'”. *(Przyznaj 2 punkty za każdą przypomnianą pozycję, która nie została podpowiedziana - użyj tylko "herbaty" jako zachęty).*		
Herbata	…….2	
Olej	…….2	
Jajka	…….2	
Mydło	…….2	
**Zasób słów**		**…./8**
6. Dam Ci teraz 1 minutę. W ciągu tej jednej minuty chciałbym, abyś wymienił mi nazwy jak największej liczby różnych zwierząt, jakie przyjdą Ci do głowy. Zobaczymy, ile różnych zwierząt potrafisz nazwać w ciągu jednej minuty.<br>(Powtórz instrukcje, jeśli to konieczne). Maksymalny wynik dla tego punktu to 8. Jeśli badany wymieni 8 różnych zwierząt w czasie krótszym niż jedna minuta, zadanie uznaje się za zakończone.<br>1. ……………………………… 5. …………………………………<br>2. ……………………………… 6. …………………………………<br>3. ……………………………… 7. …………………………………<br>4. ……………………………… 8. …………………………………		
		**…./8**
**WYNIK=**		**…./30**

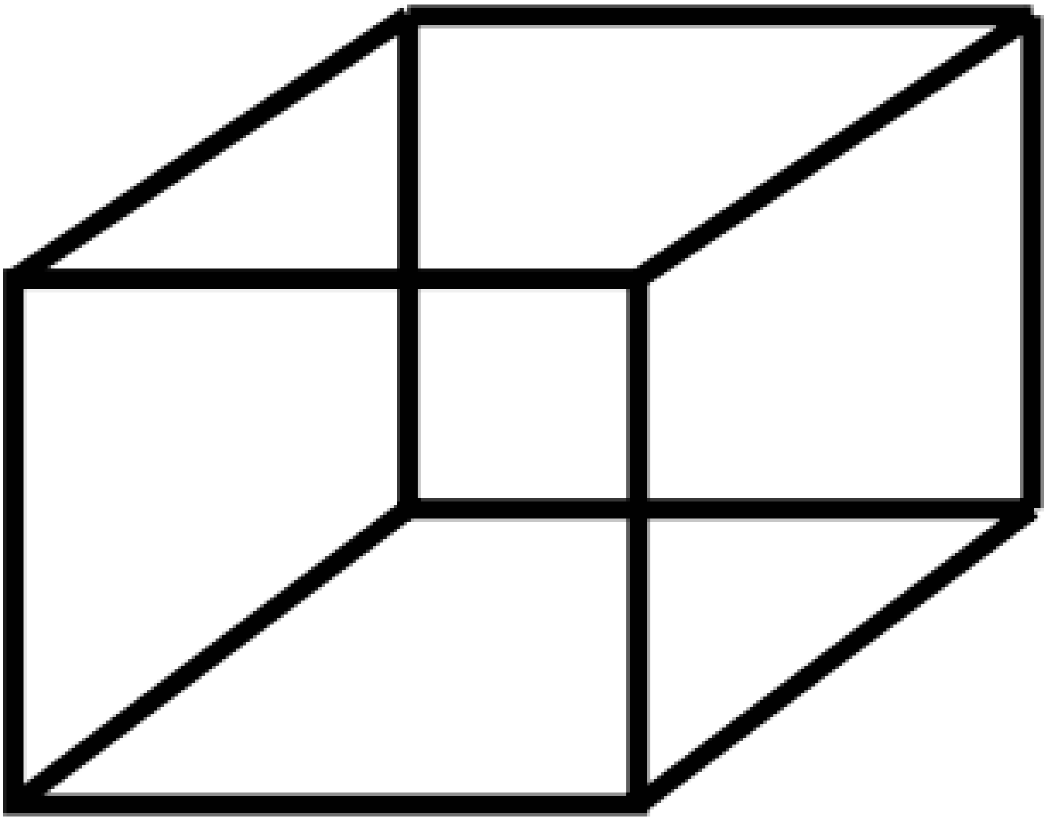

Supplement: Supplementary file 1 [file diagnostics-15-03005-s001.zip › diagnostics-3929164-supplementary.pdf]
